# Genome-Wide Gene Expression Profiles Reveal Distinct Molecular Characteristics of the Goose Granulosa Cells

**DOI:** 10.3389/fgene.2021.786287

**Published:** 2021-12-17

**Authors:** Guangliang Gao, Silu Hu, Keshan Zhang, Haiwei Wang, Youhui Xie, Changlian Zhang, Rui Wu, Xianzhi Zhao, Hongmei Zhang, Qigui Wang

**Affiliations:** ^1^ Chongqing Academy of Animal Sciences, Chongqing, China; ^2^ Institute of Animal Genetics and Breeding, College of Animal Science and Technology, Sichuan Agricultural University, Chengdu, China; ^3^ Chongqing Engineering Research Center of Goose Genetic Improvement, Chongqing, China; ^4^ Department of Cardiovascular Ultrasound and Non-invasive Cardiology, Sichuan Academy of Medical Sciences and Sichuan Provincial People’s Hospital, Chengdu, China; ^5^ Ultrasound in Cardiac Electrophysiology and Biomechanics Key Laboratory of Sichuan Province, Chengdu, China

**Keywords:** goose, ovary development, follicle selection, differentiation, proliferation

## Abstract

Granulosa cells (GCs) are decisive players in follicular development. In this study, the follicle tissues and GCs were isolated from the goose during the peak-laying period to perform hematoxylin-eosin staining and RNA-seq, respectively. Moreover, the dynamic mRNA and lncRNA expression profiles and mRNA-lncRNA network analysis were integrated to identify the important genes and lncRNAs. The morphological analysis showed that the size of the GCs did not significantly change, but the thickness of the granulosa layer cells differed significantly across the developmental stages. Subsequently, 14,286 mRNAs, 3,956 lncRNAs, and 1,329 TUCPs (transcripts with unknown coding potential) were detected in the GCs. We identified 37 common DEGs in the pre-hierarchical and hierarchical follicle stages, respectively, which might be critical for follicle development. Moreover, 3,089 significant time-course DEGs (Differentially expressed genes) and 13 core genes in 4 clusters were screened during goose GCs development. Finally, the network lncRNA G8399 with *CADH5* and *KLF2*, and lncRNA G8399 with *LARP6* and *EOMES* were found to be important for follicular development in GCs. Thus, the results would provide a rich resource for elucidating the reproductive biology of geese and accelerate the improvement of the egg-laying performance of geese.

## Introduction

Granulosa cells (GCs) not only regulate follicular development in poultry through proliferation, differentiation, apoptosis, and steroidogenesis but also provide an essential microenvironment for follicular growth by synthesizing and secreting various hormones (Johnson, 2015; Hu et al., 2017). In poultry, a highly efficient follicular development is essential for excellent egg-laying (Niu et al., 2021). Although goose is one of the economically important waterfowls, its imperfect reproductive performance seriously dampens the potential development of the goose industry (Gao G. et al., 2015). To improve reproductive performance, the molecular mechanism of GCs in regulating follicular development has been extensively studied (Liu et al., 2015). For example, some genes play important roles in goose follicular development, such as *AMH* (Anti-Mullerian Hormone) (Johnson, 2012), *FSHR* (Follicle Stimulating Hormone Receptor) (Kang et al., 2009), *CYP17* (Cytochrome P450 Family 17) (Huang et al., 2018), *Smad9* (SMAD Family Member 9) (Yu et al., 2017), and *BMP4* (Bone Morphogenetic Protein 4) (Yuan et al., 2019).

Unlike mammals, the follicles in poultry possess unique characteristics. The follicles are classified according to the size, as pre-hierarchical or hierarchical follicles. The pre-hierarchical follicles differ from the hierarchical follicles in undergoing a process of raising and selection; under normal circumstances, with no follicular selection, the number of hierarchical follicles remains unchanged (Meng et al., 2019; Shen et al., 2019). Moreover, the hierarchical follicles mature to ovulate, and eventually, when the single follicle is formed, the follicle selection is repressed (Johnson, 2015). Clear differences in the histological character and steroidogenesis between the pre-hierarchical and hierarchical follicles have been identified, conferring the physical and functional readiness for follicle development (Deng et al., 2018). Besides, significant changes in histomorphology and steroid synthesis and secretion occur during follicular development (Ocn-Grove et al., 2012). After being selected to develop into hierarchical follicles, the thickness of the granular layer is gradually decreased to a single layer, while that of the membrane layer is rapidly increased (Kim et al., 2013).

This study investigated the phenotypic differences in the size of the GCs, and the thickness of the granulosa layer cells at these developmental stages by HE staining. Then, the temporal expression profiles of the differentially expressed genes (DEGs) and differentially expressed long noncoding RNAs (DElncRNAs) were determined across the GCs, and the genes or lncRNA (Long non-coding RNA) in GCs crucial during the follicular development were screened. Furthermore, the *cis*-and *trans*-target genes were predicted and the mRNA-lncRNA network was constructed to analyze the important genes and lncRNAs. Our findings thus aim to broaden our understanding of follicle development, providing a new robust resource for gene discovery and validation in goose.

## Materials and Methods

### Experimental Animals and Sample Collection

All the animal experiments were carried out strictly according to the guidelines of the Animal Care and Welfare Committee of Chongqing Academy of Animal Science (CAAS), China. The Sichuan White goose population was reared individually in the cages (600 × 800 × 900 mm) and fed rice grains ad libitum (Wang et al., 2017) under natural environmental conditions in the waterfowl experimental base of the waterfowl-breeding base in Rongchang County, Chongqing City, China (105.48°N, 29.34°E). The egg-laying performance of the individuals was recorded and statistically analyzed twice a day. Ten individuals that consequently laid eggs during the peak egg-laying period (45 weeks) were selected as experimental animals. These geese were slaughtered 2 h before egg-laying to obtain the follicle tissue samples (SWF, LWF, SYF, LYF, F5 to F1) that were representatives of the end of each of the developmental stages. Of these ten individuals, five were used to survey the changes in the histomorphology of the follicle tissues, and the remaining five were used to obtain a dynamic gene expression atlas of the goose GCs. The follicles were classified according to the diameter and color into the small white follicle (SWF, 2–4 mm), large white follicle (LWF, 4–6 mm), the small yellow follicle (SYF, 6–8 mm), the large yellow follicle (8–10 mm, LYF), the fifth-largest follicle (F5), the fourth-largest follicle (F4), the third-largest follicle (F3), the second-largest follicle (F2), and the largest follicle (F1).

### Morphometry of the Follicular Tissues

The follicular tissues were fixed at 4°C with paraformaldehyde (4%). After 24 h of fixation, the tissues were buried in paraffin and sliced by a fully automatic dehydrator along the axes for histological staining using hematoxylin-eosin (HE). The follicle samples were analyzed using an Olympus BX51 microscope (Olympus, Tokyo, Japan) equipped with dry lenses and a microscope digital camera, Olympus DP70 (Olympus, Tokyo, Japan). After staining each slice with hematoxylin and eosin (H&E staining), the structure of the follicular tissues at 100× and 400× magnifications, including the thickness of the granular layer and the size of GCs in each stage were analyzed using the Image-Pro Plus software (version 6.0). More than five follicles at each stage were randomly selected, with more than five different regions being randomly selected from each follicle for slicing, while more than five different regions were also randomly selected from the follicles at each of F5–F1 for slicing.

### Total RNA Extraction, Library Preparation, and Sequencing

The GCs were collected from the follicular tissues in the phosphate-buffered saline (PBS) and immediately frozen in liquid nitrogen, followed by storage at −80°C for RNA-seq and real-time PCR. The total RNA was extracted using the Trizol reagent (Invitrogen, Carlsbad, CA, United States) and further purified using an RNeasy column (Qiagen, Valencia, CA, United States), according to the manufacturer’s instructions. The NanoDrop ND-1000 (Nanodrop Technologies, Wilmington, DE, United States) and 1% agarose gel electrophoresis were employed to analyze the concentration and integrity of RNA, respectively. Finally, a total of 45 total RNA samples (4 μg each) were isolated from the nine stages of the five individuals. The mRNA isolated from the total RNA by binding oligo (dT) magnetic beads was cleaved into fragments (approximately 155 bp). The 45 cDNA libraries were constructed using these fragments, as described previously (Gao G. L. et al., 2015), and then sequenced using Illumina Hiseq X Ten (Novogene Bioinformatics Technology Co., Ltd., Beijing, China; http://www.novogene.com).

### Data Analysis

After removing the adaptors, the sequences with uncertain bases, low-quality sequences, and sequences of less than 50 bp, as well as the high-quality data from the 45 libraries were mapped to the goose genome, which was assembled by our lab (Li et al., 2020) by alignment tool STAR (version 2.6.0c) (Dobin and Gingeras, 2015; Dobin and Gingeras, 2016) with the parameters of ENCODE standard RNA-seq pipeline. Then, the transcripts were reconstructed with parameters of *-g -u -b --library-type fr-first strand* using the Cufflinks (2.2.1) (Pollier et al., 2013; Ghosh and Chan, 2016). Then, transcripts were filtered by length (<250 bp), an expression level (FPKM <0.1), and clipped-exons (first or last exons <15 bp were clipped) for each library, and the Assemblyline utility (https://code.google.com/archive/p/assemblyline/) was used to filter the transfrags produced by the background noise (Iyer et al., 2015). Next, all the filtered libraries were merged and compared with the reference annotation by TACO(Niknafs et al., 2017) to remove the transcripts annotated as mRNAs. The remaining transcripts were considered to be non-coding RNAs and were subjected to lncRNA and TUCP (transcripts with unknown coding potential) identification. For the followed identification, the CPC2 (http://cpc2.cbi.pku.edu.cn/) (Kang et al., 2017) was run to analyze the coding potential, and Pfamscan (v.1.6) was applied to check the domain hits of the nucleic acid sequences against the Pfam (release31) database after the open reading frames were obtained using EMBOSS (version 6.5.7). A domain hit by mRNAs and putative lncRNAs were checked by Fisher’s exact test, and those domains with an odds ratio of less than 10.0 or *p*-value greater than 0.05 were considered as likely artifacts. After removing the transcripts in all likely domains and with coding potential (CPC score >0), the remaining transcripts were identified to be long non-coding RNAs. We classified the lncRNAs into five types (intergenic lncRNAs; antisense lncRNAs; sense-overlapping lncRNAs; divergent lncRNAs and convergent lncRNAs) as stated previously (Jin et al., 2018). To identify the expression profiles of the mRNA, lncRNA, and TUCP gene, the hierarchical clustering was performed using hclust function in R (stats package v3.5.3) (Team, 2013). Also, the dimensional reduction analysis was performed and the result was visualized using the Rt-SNE package (Van Der Maaten and Hinton, 2008).

### The Analysis of Differentially Expressed Genes (DEGs) and Cluster Analysis Time-Series

We employed the EdgeR software to analyze the DEGs between the adjacent pairs of developmental stages using the TPM normalization method. The fold changes were also estimated within this package, and the false discovery rate (FDR) was obtained by adjusting the *p*-value. A 0.05 FDR and 1.5-fold change were set as the threshold to define DEGs. Moreover, the next-maSigPro method of the maSigPro program R package was used to identify the genes with differential temporal expression profiles from SWF to F1 GCs (Nueda et al., 2014). The differentially expressed targets were classified into four clusters using the hclust function of maSigPro, and the median profiles of the resulting clusters were plotted using the maSigPro to visualize their expression patterns.

### The Prediction of the Cis-target and Trans-target Genes

The *cis*-target genes of lncRNA were defined as the mRNA located within 100 kb, whereas the *trans*-target genes were those having a Pearson correlation coefficient more than 0.95 or less than −0.95 with *p*-value < 0.05. The *cis*-target genes and *trans*-target genes of lncRNA, and the four gene expression patterns were integrated. Then, the co-expression mRNA-lncRNA network was generated by analyzing using the weighted gene co-expression network analysis (WGCNA) (Langfelder and Horvath, 2008). The genes with the number of edges equal or more than ten were defined as the hub genes, and then the networks were visualized by the Cystoscope software (version 3.6.0) (Kohl et al., 2011).

### Quantitative Real-Time PCR

To confirm the results of the RNA-seq, six of the DEGs were randomly selected for qRT-PCR. The total RNAs were isolated from the same samples used for the RNA-seq of the 45 individuals. The goose glyceraldehyde-3-phosphate dehydrogenase (*GAPDH*) gene was selected as an internal control gene for normalization. The primers of the six genes were designed for qRT-PCR analyses (Supplementary Table S1) and synthesized by Invitrogen (Shanghai, China). Three independent qPCR runs were performed using the Applied Biosystems 7900 HT Sequence Detection System (Applied Biosystems, Foster City, CA, United States) in 20 μl reaction mixtures using the SYBR Premix Ex Taq system (Takara, Co., Dalian, China). The following qPCR program was applied: a single cycle of 95°C for 1 min; 40 cycles of 95°C for 5 s and 60°C for 34 s; and one cycle for analyzing the melting curve analysis. The relative expression levels of the candidate genes relative to *GAPDH* (Glyceraldehyde-3-Phosphate Dehydrogenase) were calculated using the 2^−ΔΔCt^ method (Livak and Schmittgen, 2001).

## Results

### Morphometry of the Follicular Tissues

**FIGURE1:**
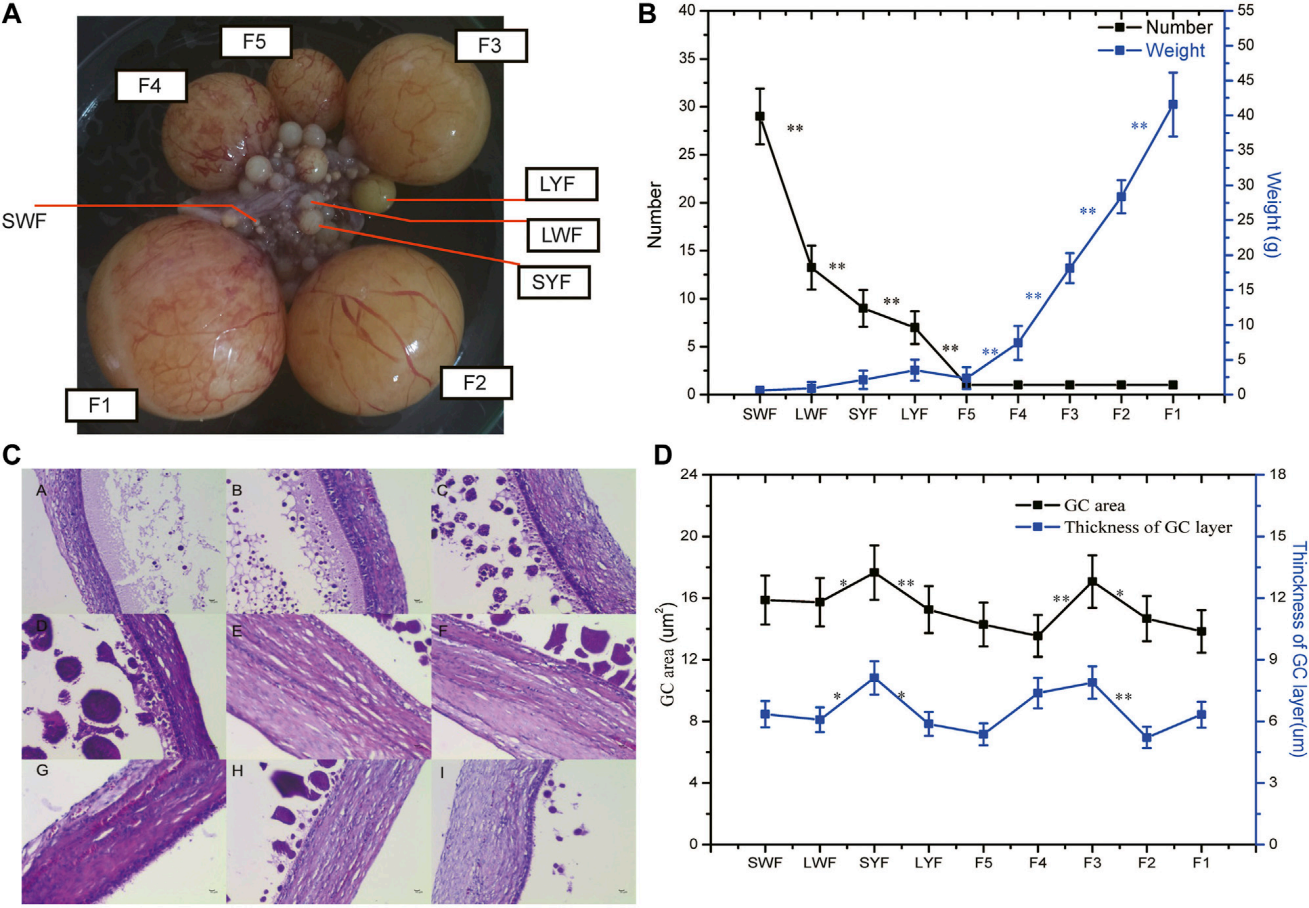
The weights and phenotypic differences in the various sizes of the GCs. Note **(A)**, The phenotypic of goose follicles; **(B)**, The numbers and weight of the goose follicles; **(C)**, Histological staining for follicular tissues using hematoxylin-eosin (HE); **(D)**, The granular cell area and thickness of the granulosa layer cells.

In this study, five sustainable egg-laying Sichuan White goose (three consecutive laid eggs in a week) during the peak-laying period (45 weeks) were selected as experimental animals (Figure 1A). Our results revealed that the number of pre-hierarchical follicles gradually decreased over time, showing extremely significant differences between the SWF vs. LWF, LWF vs. SYF, SYF vs. LWF, and LWF vs. F5, respectively (*p* < 0.01) (Figure 1B). There was a single follicle for the hierarchical follicles (F5–F1) during every developmental stage, but the weight gradually increased from SWF to F1 (Figure 1B). The HE staining results showed that the granular cell area of the nine follicles (Figure 1C) showed no differences between any developmental stages. However, the thickness of the granulosa cell layer increased and then decreased to its nadir in the LYF follicles (Figure 1D).

### Data Statistics

A total of 315.98 Gb raw reads were obtained from these 45 libraries ranging from 4.74 to 9.74 Gb. After quality control, we acquired 311.84 Gb clean data (∼6.93 Gb per each) of all samples ranging between 4.68 and 9.63 Gb, and the qualities and alignment ratios were shown in Supplementary Table S2. In this study, the 8,199 lncRNA transcripts (from 5,531 gene locus) were identified, of which 7,045 were classified into intergenic lncRNAs; antisense lncRNAs; sense-overlapping lncRNAs; divergent lncRNAs, and convergent lncRNAs (Supplementary Figure S1). Besides, we identified 2,664 TUCPs (from 1,444 gene locus).

**FIGURE 2:**
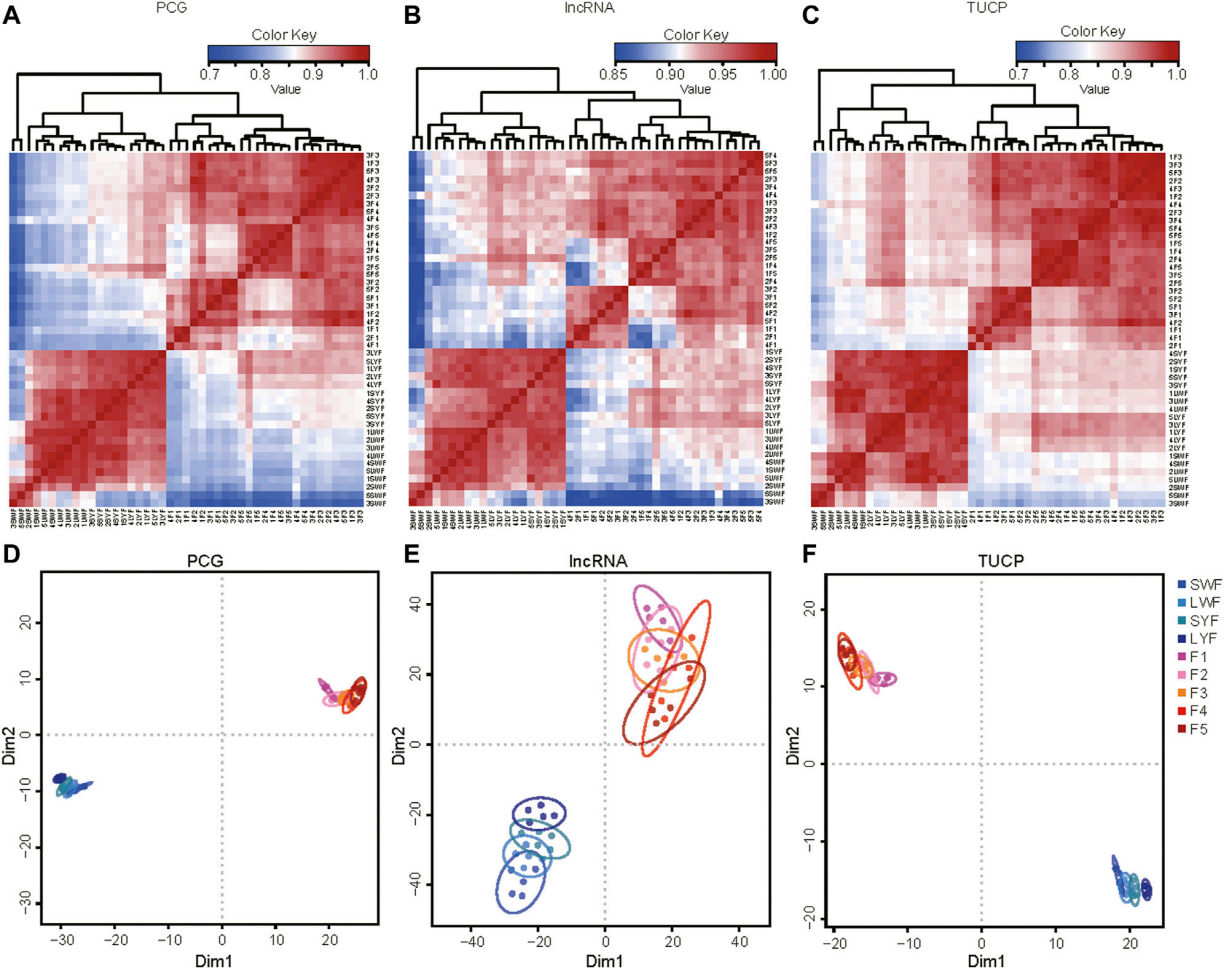
The common DEGs in the pre-hierarchical follicles and hierarchical follicle GCs.

Then, the expression level of PCG, lncRNA, and TUCP were normalized to TPM (Transcripts Per Kilobase of exon model per Million mapped reads) with the software Kallisto (Bray et al., 2016), and PCG with TPM >0.5 in at least three replicates of one group, the lncRNA or TUCP with TPM >0.1 in at least three replicates of one group were considered to be expressed and were subjected to the continuing analysis. In total, 14,286 mRNAs, 3,956 lncRNAs, and 1,299 TUCPs were substantially expressed in our research. Furthermore, the results of Pearson matrix correlations of mRNA (Figure 2A), lncRNA (Figure 2B) and TUCP (Figure 2C), and t-SNE of mRNA (Figure 2D), lncRNA (Figure 2E) and TUCP (Figure 2F) showed that the 45 samples could be differentiated at both PCG, lncRNA, and TUCP of GCs in the expression levels of the pre-hierarchal follicles and hierarchal follicles.

**FIGURE 3:**
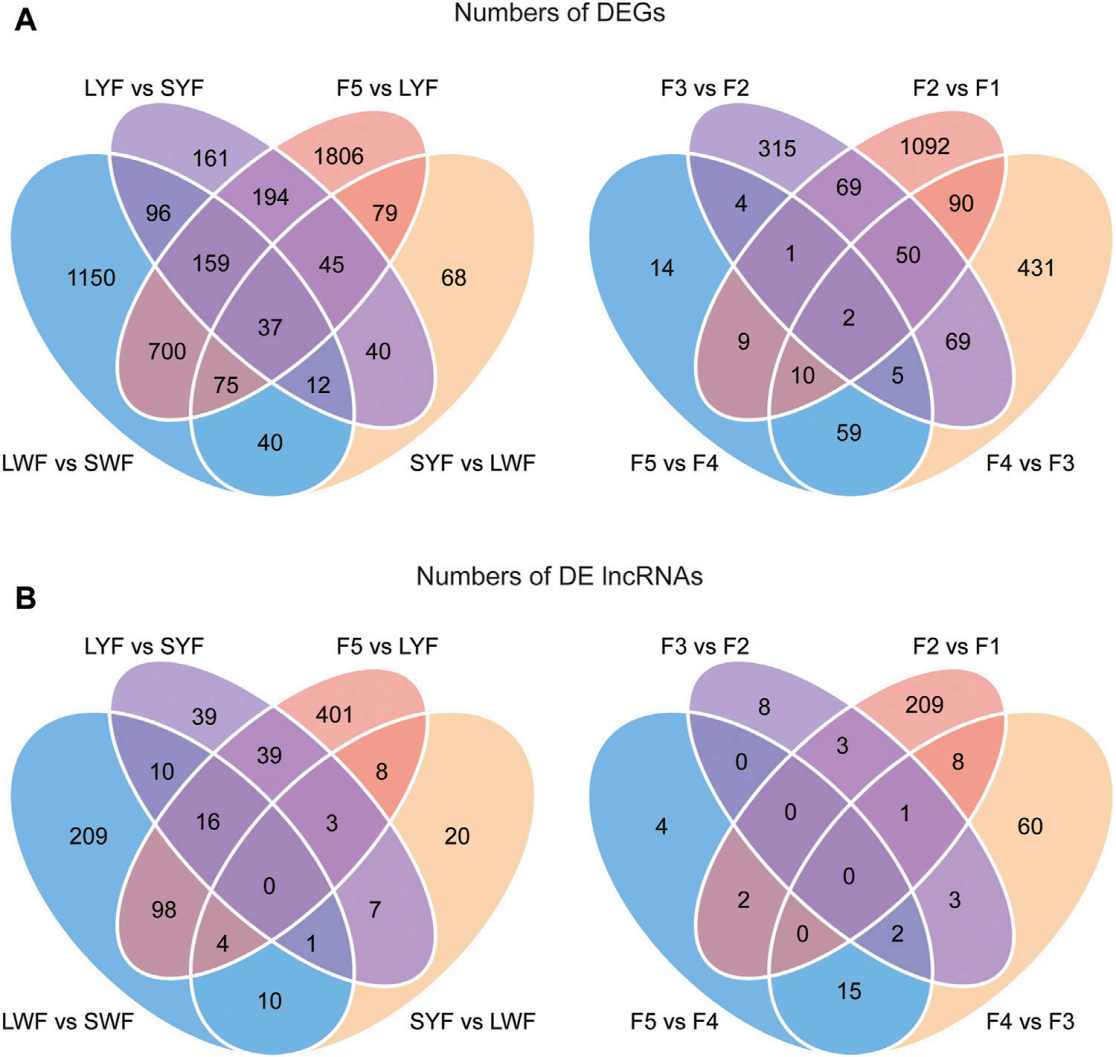
The common DEGs and DElncRNAs detected by comparing the adjacent pairs of developmental stages. Note **(A)**: The common DEGs in the pre-hierarchical follicles and hierarchical follicle GCs; **(B)**: The common DElncRNAs in the pre-hierarchical follicles and hierarchical follicle GCs.

In this study, 2,269, 396, 744, 3,095, 104, 716, 515, and 1,323 DEGs (Supplementary Table S3) were detected by comparing the adjacent pairs of developmental stages (i.e., SWF vs. LWF, LWF vs. SYF, SYF vs. LYF, LYF vs. F5, F5 vs. F4, F4 vs. F3, F3 vs. F2, and F2 vs. F1 groups, respectively). To investigate the important DEGs from the SWF to F1 developmental stages, we screened the common DEGs with vital roles in regulating the follicular developmental stage. The results revealed 37 and 2 genes commonly and differentially expressed in the pre-hierarchical and hierarchical follicles (Figure 3A), suggesting that the 37 and 2 genes be important for the pre-hierarchical GCs (SWF, LWF, SYF, and LYF) and hierarchical GCs (F5, F4, F3, F2, and F1), respectively.

In this study, 348, 53, 115, 569, 22, 88, 16, and 223 DElncRNAs were also identified between the adjacent stages (SWF vs. LWF, LWF vs. SYF, SYF vs. LYF, LYF vs. F5, F5 vs. F4, F4 vs. F3, F3 vs. F2, and F2 vs. F1 groups, respectively) (Figure 3A; Supplementary Table S4). We found no common DElncRNAs in the pre-hierarchical follicles or hierarchical follicles (Figure 3B). To evaluate the accuracy of the results from RNAseq, the quantitative real-time PCR method was employed to detect the genes expression patterns of *WASL* (Wiskott-Aldrich syndrome protein), *GPR63* (G-protein coupled receptor 63), *FST* (Follistatin precursor), *AtTTP* (At1g68200), *NNT* [NAD(P) transhydrogenase, mitochondrial], and *P2RY6* (Pyrimidinergic Receptor P2Y6) genes (Supplementary Figure S2). The results were consistent with the RNA-seq data, demonstrating that the results were robust and credible.

### Cluster Analysis Time-Series Analysis

**FIGURE 4:**
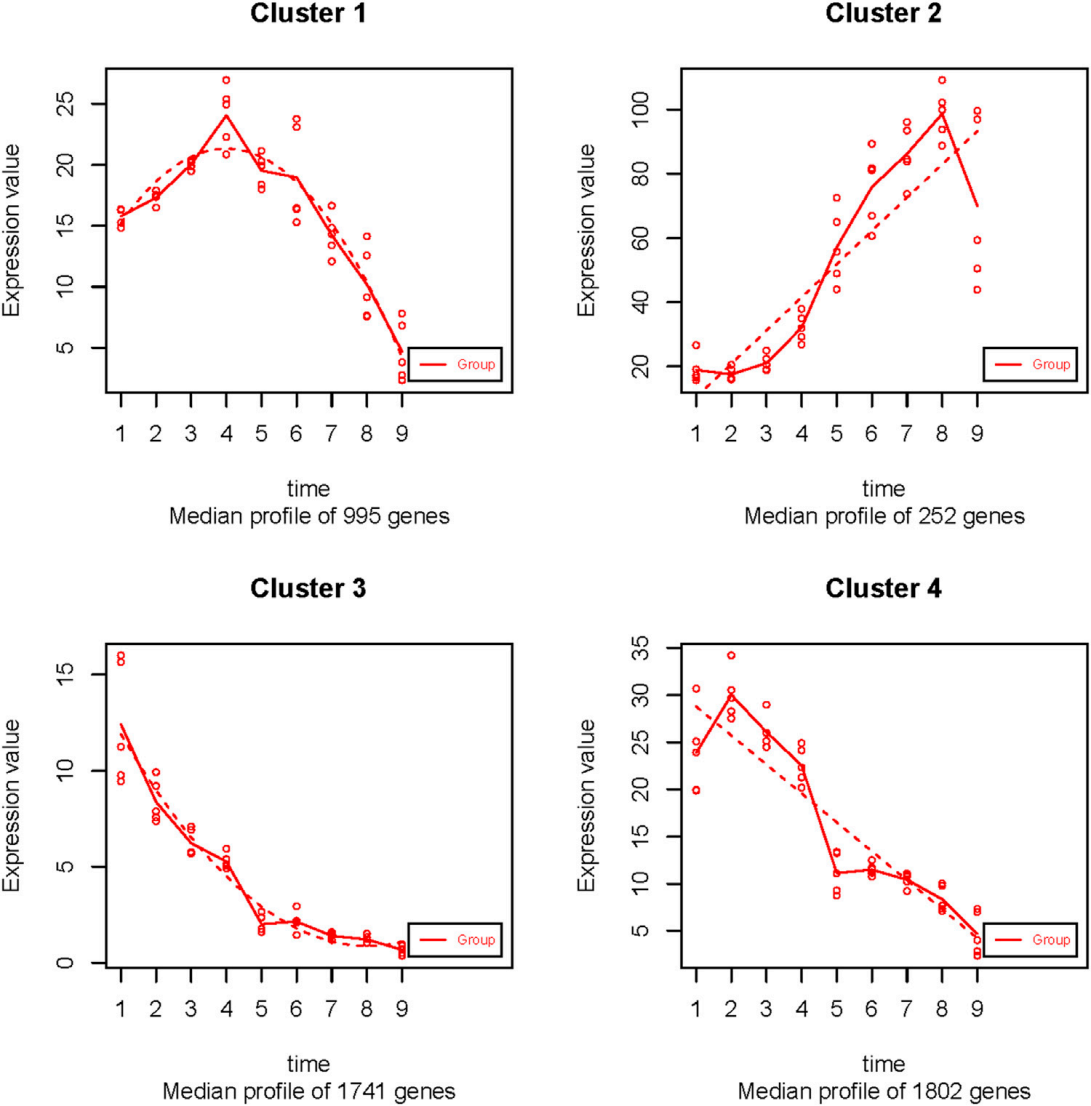
The 4 temporal gene expression profiles.

To investigate the genome-wide mRNA and lncRNA expression pattern profiles during the development of the follicular GCs, the nine-stage (SWF, LWF, SYF and LYF, F5 to F1) of goose GCs were considered with temporal changes to determine the progression patterns, similar to that of the GCs development. The maSigPro method was employed to identify 3,089 significant time-course DEGs (Supplementary Table S5) and were divided into 4 clusters at the nine-time point of GCs (Figure 4; Supplementary Figure S3).

Overall, the 995 gene expression in cluster 1 reached the top point in LYF, and then consistently decreased to the lowest point at the SWF stage (Figure 4). The expression of 252 genes in cluster 2 was relatively lower in the pre-hierarchical period than that in the hierarchical period, which consistently increased and reached the peak in the F2 GC stage (Figure 4). On the contrary, the expression of 1741 genes in cluster 3 was similar to that of cluster 4, containing 1802 genes, which were expressed at a comparatively higher level during the pre-hierarchical period, and drooped to the bottom in the F1 GC stage, but the difference between the two clusters was that the top of genes expression at SWF in the cluster 3, rather than that in the cluster 4 in LWF (Figure 4).

**FIGURE 5:**
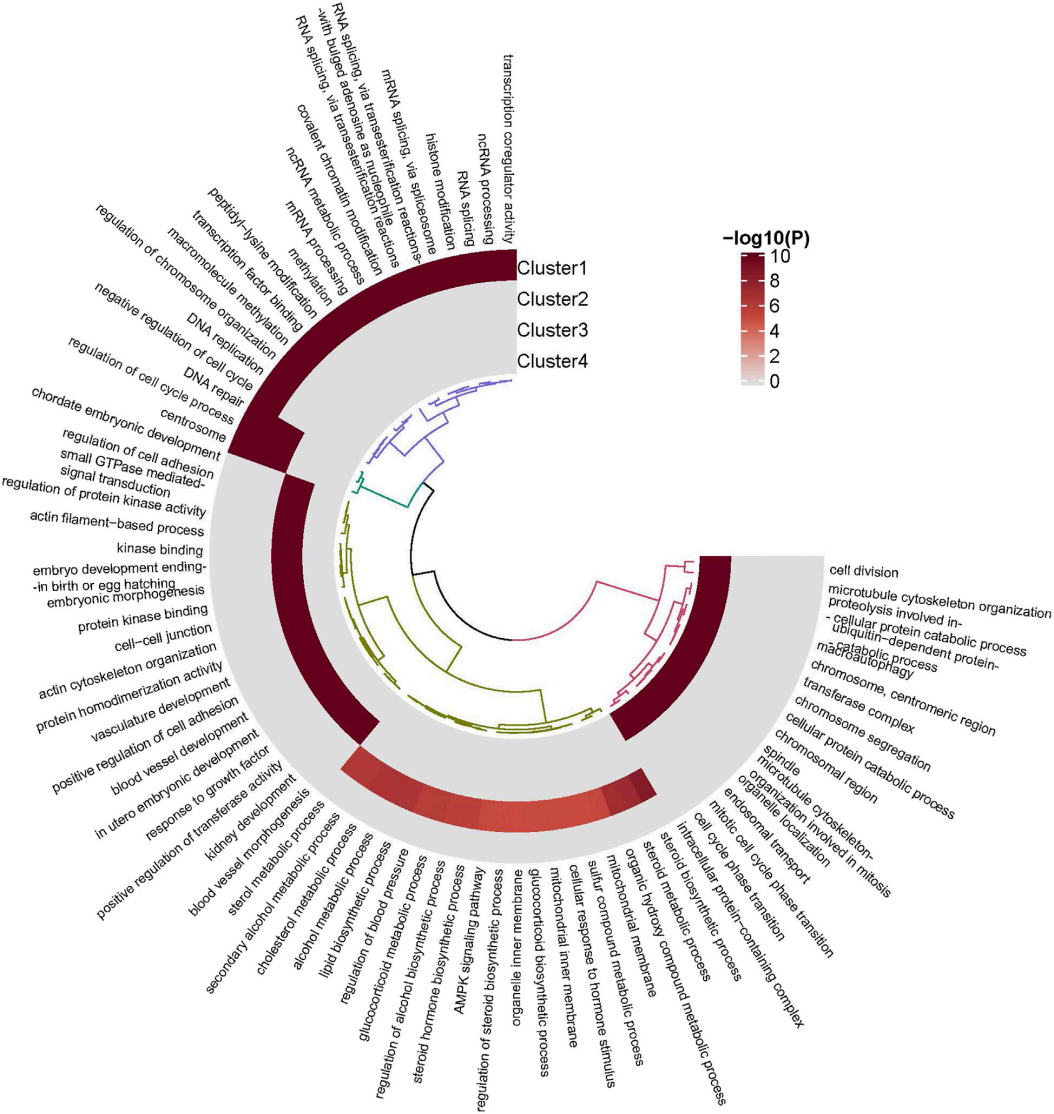
The gene enrichment for the 4 clusters.

The gene functional enrichment analysis was performed by the Metascape website to investigate the biological characteristics of the genes in the 4 clusters during the GC development. 1) The gene function analysis showed that the genes in cluster 1 were significantly enriched in mRNA processing (GO:0006397), ncRNA metabolic process (GO:0034660), covalent chromatin modification (GO:0016569). 2) The result of functional enrichment showed that the genes in cluster 2 significantly enriched in the steroid biosynthetic process (GO:0006694), steroid metabolic process (GO:0008202), organic hydroxy compound metabolic process (GO:1901615). 3) The functional analysis showed that the genes in cluster 3 were significantly enriched in the regulation of protein kinase activity (GO:0045859), regulation of cell adhesion (GO:0030155), regulation of cell adhesion (GO:0043009). 4) The results of the functional enrichment showed that the genes in cluster 4 were significantly enriched in cell division (GO:0051301), microtubule cytoskeleton organization (GO:0000226), mitotic cell cycle phase transition (GO:0044772). The DNA repair (GO:0006281), regulation of cell cycle process (GO:0010564), the centrosome (GO:0005813), cell projection assembly (GO:0030031), plasma membrane-bounded cell projection assembly (GO:0120031) were common in clusters 1 and 2 (Figure 5).

### Core Genes Screening

**FIGURE 6:**
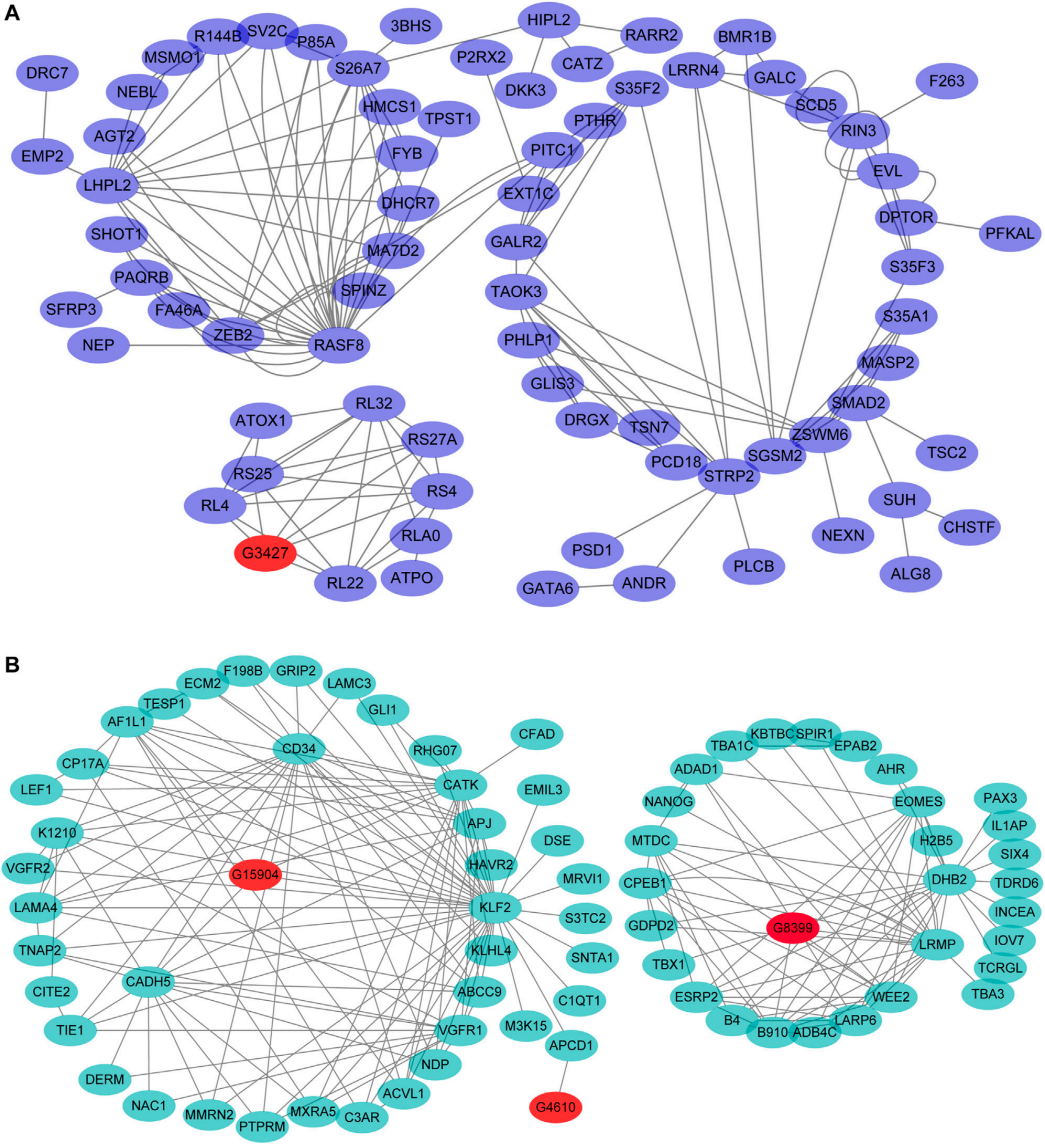
The *cis*-and *trans*-target genes were predicted and the mRNA-lncRNA network in cluster 2 and 3. Note: **(A)**, The mRNA-lncRNA network in cluster 2; **(B)**, The mRNA-lncRNA network in cluster 3.

Thirteen hub genes were screened from the four clusters (Figure 6; Supplementary Figure S4). There were three hub genes in the cluster1: *GON4L* (*GON-4-like protein*), *NUMA1* (*Nuclear mitotic apparatus protein 1*), *SAFB1* (*scaffold attachment factor B1*) (Supplementary Figure S4); In cluster2, the core gene was *RASF8* (*Ras association domain-containing protein 8*) (Figure 6). There were six core genes in the cluster3: *APJ* (*Apelin receptor*), *CATK* (*Cathepsin K*), *CD34* (*Hematopoietic progenitor cell antigen CD34*), *EOMES* (*Eomesodermin*), *KLF2* (*Krueppel-like factor 2*), *VGFR1* (*Vascular endothelial growth factor receptor 1*) (Figure 6). There were three genes in the cluster4: *PLPL7*(Patatin-like phospholipase domain-containing protein 7), *RFA2* (Replication Protein A2), and *TMOD1* (Tropomodulin-1) (Supplementary Figure S4).

### Exploration of the Genes That Continuously Regulated the Development of GCs in Goose

It is difficult to predict the function of the three types of non-coding RNAs owing to the lacing of the annotation for lncRNAs in goose. Here, we explored the functional relatedness between the mRNAs and *cis*-lncRNAs or *trans*-lncRNAs using the co-expression analysis or the WGCNA method in the 4 clusters. In cluster 2, the G3427 co-expressed with *RL4* (Ribosomal Protein L4), *RS25* (Ribosomal Protein S25), *RL32* (Ribosomal Protein L32), *RS27A* (Ribosomal Protein S27a), *RS4* (Ribosomal protein S4), and *RL22* (Ribosomal Protein L22) (Figure 6). In cluster 3, the G15904 co-expressed with *CADH5* (Cadherin 5), *KLF2* (Kruppel Like Factor 2), *CD34* (CD34 Molecule), and the G4610 were associated with *APCD1* (APC Down-Regulated 1). The G8399 co-expressed with *ESRP2* (Epithelial Splicing Regulatory Protein 2), *ADAD1* (Adenosine Deaminase Domain Containing 1), *EOMES* (Eomesodermin), *DHB2* (Estradiol 17-beta-dehydrogenase 2), *LRMP* (Lymphoid-restricted membrane protein), and *LARP6* (La Ribonucleoprotein 6, Translational Regulator) and *B910* (Maternal B9.10 protein) genes (Figure 6).

## Discussion

### Morphometry of the Follicular Tissues

The pre-hierarchal and preovulatory follicles clusters in goose are subjected to stages like initiation, development, and selection. The number of follicles in the goose was found to decrease from SWF to LYF, while the diameters and the weights increased simultaneously (Figure 1A), which is consistent with that of the other poultry, (Johnson, 2015; Gao et al., 2019). These results suggested that follicle selection occurs in the pre-hierarchical follicles rather than in the hierarchical ones, and the number of hierarchical follicles instead remains unchanged suppressing the follicle selection consistent with that in other poultry, along with the increase in the follicular diameters and weight (Ghanem and Johnson, 2018). Moreover, the thickness of the granulosa cell layer was found to increase and then decrease in the LYF follicles (Figures 1C,D), which were consistent with the findings of a previous study on the histological study of the pre-hierarchical follicles in goose (Dong et al., 2014).

### The DEGs and DElncRNAs Between the Adjacent Pairs of Developmental Stages

The PCA analysis showed that the nine GCs groups were clustered into two groups: pre-hierarchical and hierarchical follicles (Figure 2). These results suggested a high correlation between the gene expression levels of the biological replicates and showed the excellent reliability of our experiment. Interesting, there were most DEGs in the LYF vs. F5 (3,095) and most DElncRNAs 569) (Supplementary Tables S3, S4). However, no differences were identified in the granular cell area or the thickness of the granulosa cell layer between LYF and F5, or F5 and F4, suggesting that the F5 developmental stage were likely to show independent and unstable transition (Onagbesan et al., 2009; Kim et al., 2016; Kim et al., 2018). Some of these genes (Figure 3), such as the *FST* gene, have multiple roles in follicular development, steroid hormone synthesis, and granulose cell proliferation (Liu et al., 2015); these genes also included the *WASL* gene, which modulates the Wnt signaling and is necessary for the hair follicle cycling in mice (Lyubimova et al., 2010).

### Cluster Analysis Time-Series Analysis

The growth and proliferation of the GCs from the pre-hierarchical follicles are the key to initiation (Eppig, 2001), and the apoptosis of GCs leads to follicular atresia (Rolaki et al., 2005; Manabe et al., 2008). The common feature in cluster 1, cluster 3, and cluster 4 are that the genes were consistently increased in the pre-hierarchical stags (including SWF, LWF, SYF, and LYF) (Figure 4; Supplementary Table S6), and the genes expression showed a continuous decline in the hierarchical stages (F5, F4, F3, F2, and F1). Previous studies demonstrated that the GCs have crucial roles in follicular development. Interestingly, the genes belonging to cluster 1 were significantly enriched in the cell differentiation pathways clusters, regulation of cell cycle process (GO:0010564), DNA replication (GO:0006260), cell division (GO:0051301), regulation of telomere maintenance (GO:0032204) (Supplementary Table S6). 2) Functional analysis showed that the genes in cluster 3 were significantly enriched in the embryonic development clusters (Supplementary Table S6), such as the chordate embryonic development (GO:0043009), urogenital system development (GO:0001655), cell morphogenesis involved in differentiation (GO:0000904), cell division (GO:0051301), gland development (GO:0048732). 3) The results of the functional enrichment showed that the genes in cluster 4 were significantly enriched in the cell division clusters (GO:0051301), autophagy clusters (GO:0006914), apoptotic signaling pathway clusters (GO:0097190) (Supplementary Table S6). These results have shown that the GCs regulated the follicular development and selection process through the genes from the pre-hierarchical follicular GCs enriched in the processes of proliferation and apoptosis, embryonic development. However, the detailed molecular mechanisms still needed further investigations.

### The Core Genes in GC Development

The hub genes identified in cluster 1 were *Gon4l*, *NUMA1, and SAFB1* (Supplementary Figure S4A). *Gon4l* was conserved within the animal species (Tsai et al., 2020), with crucial roles in cell proliferation, cell differentiation, cell cycle, cell viability, embryonic patterning, cardiomyocyte proliferation, and ventricular fate maintenance in worms, flies, mice, and fish (Friedman et al., 2000; Liu et al., 2007; Bulchand et al., 2010; Lu et al., 2010; Colgan et al., 2021). Moreover, *Gon4l* has been reported to participate in cell proliferation, cell survival, and mesoderm-derived tissue specification including those of the blood and somites in the zebrafish, (Budine et al., 2020). Deficiency of *Gon4l* in zebrafish results in cellular apoptosis and failure of the cell to differentiate, blocking the mitotic cell division in the developing embryos (Friedman et al., 2000; Liu et al., 2007). One of the hub genes in cluster1, *NUMA1*, is involved in the maintenance and formation of the spindle poles and mitotic spindle organization during meiotic cell division.(Chu et al., 2016; Torii et al., 2020). The absence of *NUMA1* resists cell death in the PTC-318 cell (Gisler et al., 2020), and the alternative splicing of the gene is associated with the cellular proliferation and centrosome amplification in the epithelial cells (Sebestyén et al., 2016). *SAFB1* regulates RNA processing and neuronal function rendering the chromatin permissive for DNA damage signaling and myogenic differentiation (Hernandez-Hernandez et al., 2013; Rivers et al., 2015). Previous studies have shown the *SAFB1* absence in mice to reduce the mice growth by affecting the IGF-1 level, affecting the reproductive ability of the female mice by reducing the levels of estradiol and progesterone. On the contrary, *SAFB1* overexpression tends to shorten the S phase of the cell cycle, thereby accelerating cell apoptosis (Townson et al., 2004; Ivanova et al., 2005). In conclusion, these three genes have vital roles in embryonic development and are involved in multiple roles in goose GCs development.

In cluster 2, *RASSF8* is provital for embryonic development in *Drosophila*, where it is involved in pupal wing cell hexagonal packing promoting wing elongation and epithelial ordering (Chan et al., 2021). RASSF8 plays a role in cell growth, regulation of the Wnt and NFκB pathways, and cell-cell adhesion (Lock et al., 2010). Moreover, the RASSF8 overexpression has been found to affect the process of cell growth, cell apoptosis (Karthik et al., 2018).

*VEGFR1,* in cluster 3, is vital for angiogenesis, blood vessel patterning on the retinal astrocytes, neuronal precursors during fetal and retinal development (Chappell et al., 2019; Marini et al., 2019). One of the main functions of *CatK* is to regulate bone resorption in mammals (Dai et al., 2020), Previous studies have shown *CatK* to be associated with diseases, including skeletal diseases, renal disease, cardiovascular system, central nervous system, respiratory system, autoimmune diseases. (Bühling et al., 2004; Yang et al., 2008; Hua et al., 2015; Zhao et al., 2019; Dai et al., 2020; Dauth et al., 2020). Moreover, *CatK* is also associated with thyroid development, brain development, lipid homeostasis, and metabolism (Dauth et al., 2020).

In cluster 4, *TMOD1* critically mediates the actin dynamics, participating in the cell migration, motility, proliferation, cycle, morphology, neurite outgrowth, and spine formation (Fischer et al., 2003; Moyer et al., 2010; Fath et al., 2011; Lu et al., 2020). *TMOD1* deficiency in mice is known to lead to embryonic lethality with cardiac defects (Fritz-Six et al., 2003). In conclusion, we speculate that *TMOD1 still* play an important role during goose GCs development.

### The Core Genes and lncRNA in GC Development

In cluster 3, the lncRNA G15904 co-expressed with *CADH5*, *CD34* (Figure 6B). Interestingly, *CADH5* has a multi-functional biological function in the endothelial cells, such as regulating cell proliferation, apoptosis, cell survival, cell adhesion, cell shape, cell motility (Dejana and Vestweber, 2013; Sauteur et al., 2014; Grimsley-Myers et al., 2020). The CDH5-null mice have severe cardiovascular defects resulting in premature embryonic death (Carmeliet et al., 1999). Moreover, another gene associated with the lncRNA, *KLF2,* is essential for the development and differentiation of the lungs (Anderson et al., 1995; Maity et al., 2020), inhibiting the endothelial cell apoptosis and promoting metabolic quiescence, thus, resulting in embryonic hemorrhage and death in the *KLF2* null mice (Doddaballapur et al., 2015). Taken together, the lncRNA G15904 may regulate the expression of the genes *CADH5* and *KLF2* by participating in the granulosa cells in embryonic development, cell proliferation, and apoptosis. However, further investigations are required for an in-depth understanding of the molecular mechanisms for the lncRNA G15904in the future.

*LARP6*, one of the co-expressed genes associated with the lncRNA G8399 encodes a protein that prevents cell death until it is triggered by dephosphorylation and degraded (Sheel et al., 2020). We also found that *EOMES,* one of the target genes for the lncRNA G8399, is involved in the trophoblast differentiation as well as gastrulation, regulating both the mesodermal delamination, endodermal specification, which are essential for developing the tissues extraembryonic to the uterus during the mammalian embryogenesis (Kidder and Palmer, 2010; Probst and Arnold, 2017). Thus, the lncRNA G8399 may participate in the cell death, development, and reproduction process, but the gene function of this lncRNA demands further investigations.

In conclusion, this study measured the expression of the genes in the nine stages of development of goose GCs. Furthermore, the genome-wide mRNA and lncRNA expression pattern profiles were investigated during the development of the follicular GCs and the function of the genes of four clusters was explored during the nine developmental stages. Furthermore, two lncRNA-gene regulatory networks were also identified in goose GCs that could be explored in the future.

## Data Availability

These high-quality datasets have been deposited in NCBI’s Gene Expression Omnibus (GEO) and are accessible through GEO Series accession number GSM3927138 to GSM3927181 (the detailed information is listed in Supplementary Table S2).
