# Supplementary figures and images for: Genome-Wide Gene Expression Profiles Reveal Distinct Molecular Characteristics of the Goose Granulosa Cells
Source: Front Genet. 2021 Dec 17;12:786287. doi: 10.3389/fgene.2021.786287 (PMC8725158; doi:10.3389/fgene.2021.786287)

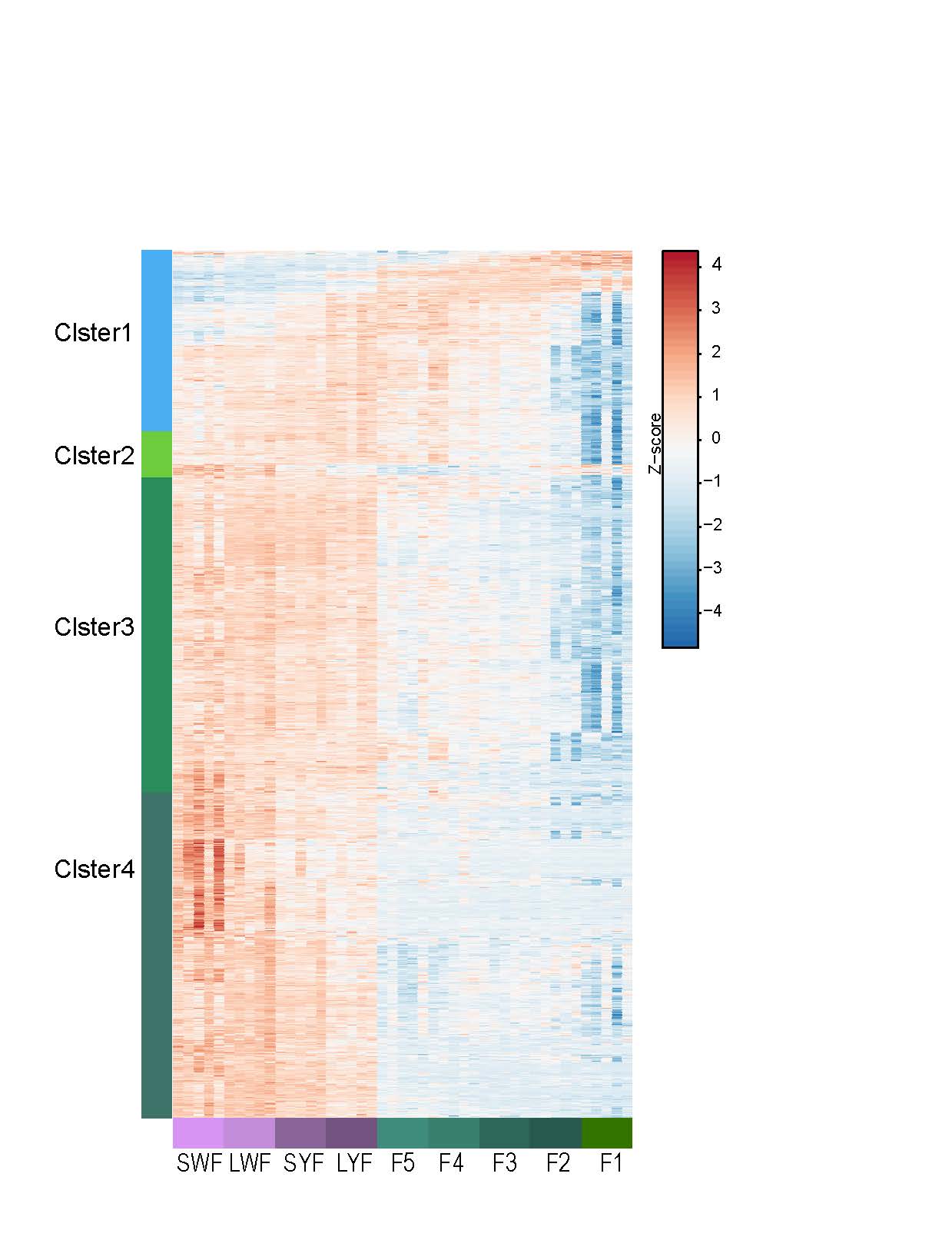

Supplement: Supplementary file 2 [file Image3.JPEG]

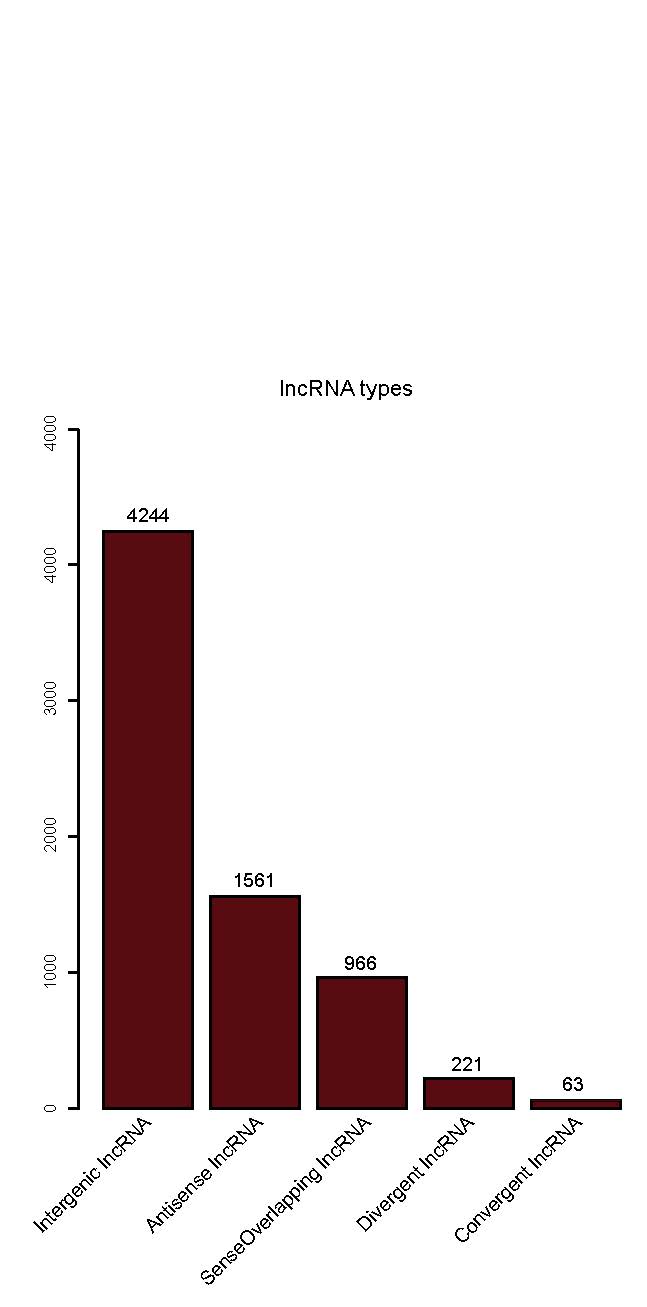

Supplement: Supplementary file 4 [file Image1.JPEG]

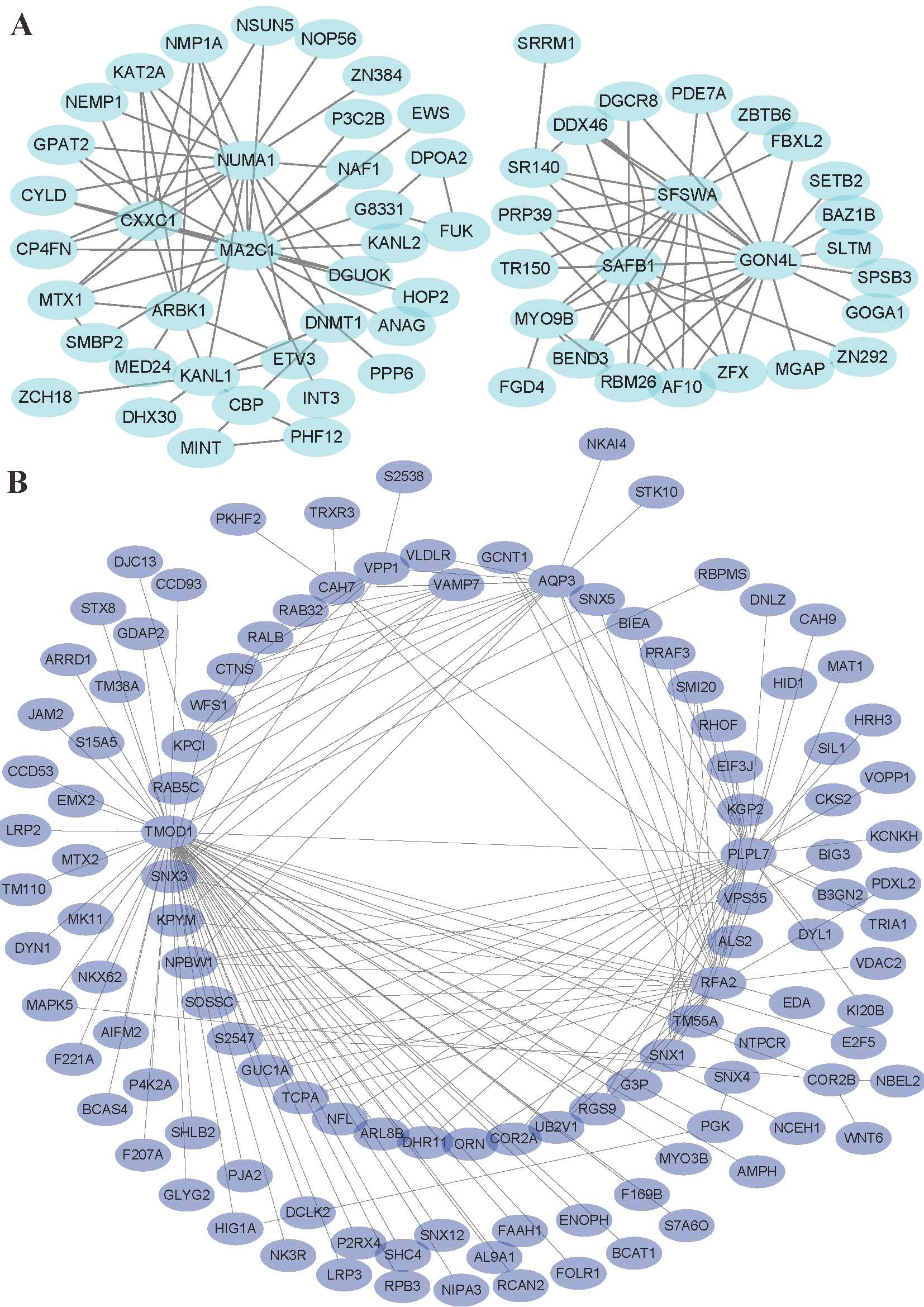

Supplement: Supplementary file 5 [file Image4.JPEG]

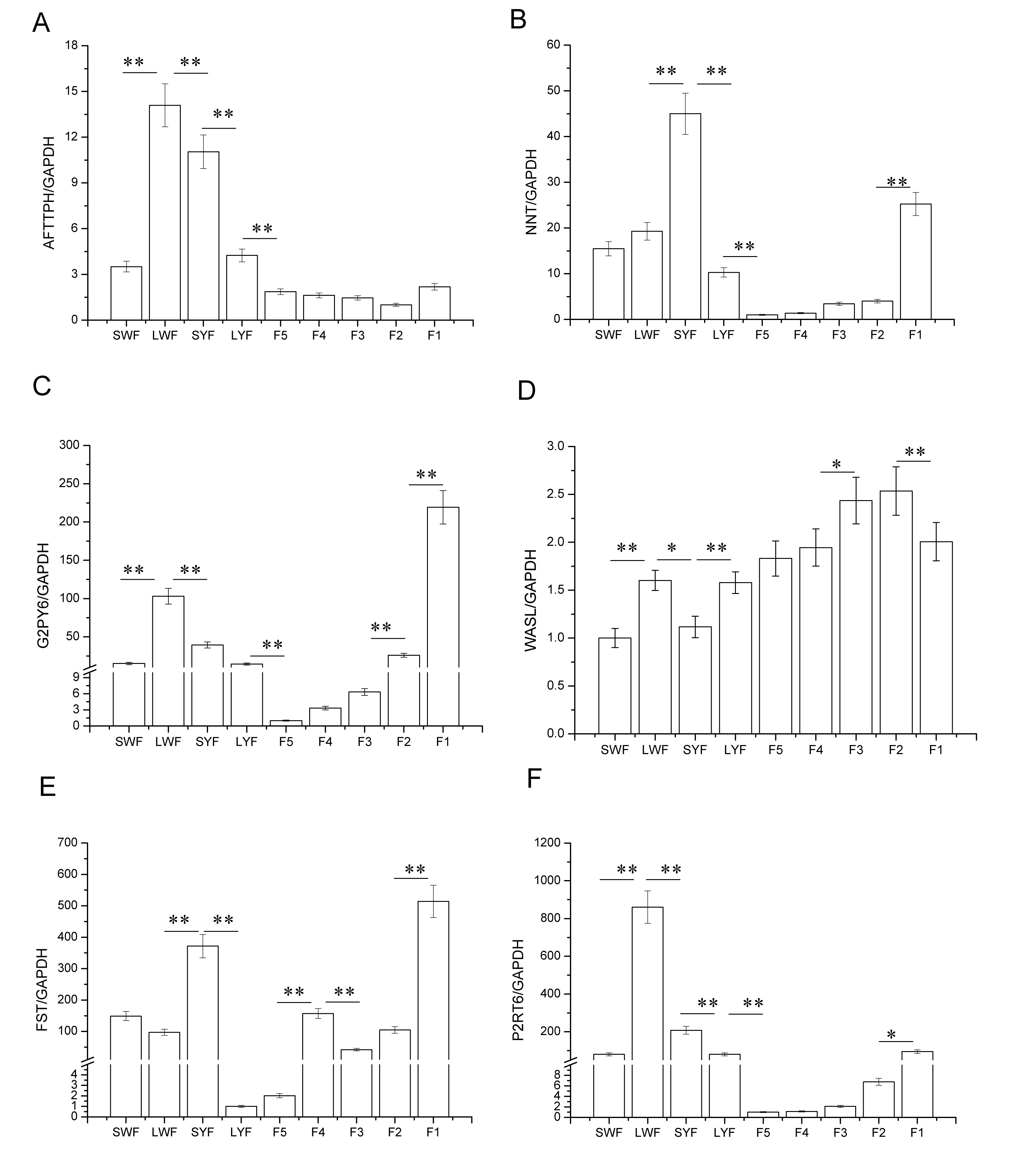

Supplement: Supplementary file 6 [file Image2.JPEG]
